# Plasma Aldosterone Concentration Is Positively Associated With Pulse Pressure in Patients With Primary Hypertension: Erratum

**DOI:** 10.1097/01.md.0000465971.09100.2d

**Published:** 2015-05-29

In the article “Plasma Aldosterone Concentration Is Positively Associated With Pulse Pressure in Patients With Primary Hypertension”,^1^ which appeared in Volume 94, Issue 10 of *Medicine*, an acknowledgment of funding was omitted. The acknowledgments section should have read as follows:

This study was supported by National Natural Science Foundation of China (Grant No. 81260129) and Special Project of Scientific and Technological Achievements Conversion of Xinjiang Uygur autonomous region (201154126).

The article has since been corrected online.
